# Testing the activitystat hypothesis: a randomised controlled trial

**DOI:** 10.1186/s12889-016-3568-x

**Published:** 2016-08-30

**Authors:** S. R. Gomersall, C. Maher, C. English, A. V. Rowlands, J. Dollman, K. Norton, T. Olds

**Affiliations:** 1 School of Health Sciences, Alliance for Research in Exercise, Nutrition and Activity, Sansom Institute for Health Research, University of South Australia, Adelaide, Australia; 2 School of Human Movement and Nutrition Sciences, Centre of Research on Exercise, Physical Activity and Health (CRExPAH), The University of Queensland, Brisbane, Australia; 3 Diabetes Research Centre, University of Leicester, Leicester General Hospital, Leicester, UK; 4 NIHR Leicester-Loughborough Diet, Lifestyle and Physical Activity Biomedical Research Unit, Leicester, UK

**Keywords:** Physical activity, Energy expenditure, Accelerometry, Compensation

## Abstract

**Background:**

It has been hypothesised that an ‘activitystat’ may biologically regulate energy expenditure or physical activity levels, thereby limiting the effectiveness of physical activity interventions. Using a randomised controlled trial design, the aim of this study was to investigate the effect of a six-week exercise stimulus on energy expenditure and physical activity, in order to empirically test this hypothesis.

**Methods:**

Previously inactive adults (*n* = 129) [age (mean ± SD) 41 ± 11 year; body mass index 26.1 ± 5.2 kg/m^2^] were randomly allocated to a Control group (*n* = 43) or a 6-week Moderate (150 min/week) (*n* = 43) or Extensive (300 min/week) (*n* = 43) exercise intervention group. Energy expenditure and physical activity were measured using a combination of accelerometry (total counts, minutes spent in moderate to vigorous physical activity) and detailed time use recalls using the Multimedia Activity Recall for Children and Adults (total daily energy expenditure, minutes spent in moderate to vigorous physical activity) at baseline, mid- and end-intervention and 3- and 6-month follow up. Resting metabolic rate was measured at baseline and end-intervention using indirect calorimetry. Analysis was conducted using random effects mixed modeling.

**Results:**

At end-intervention, there were statistically significant increases in all energy expenditure and physical activity variables according to both accelerometry and time use recalls (*p* < 0.001) in the Moderate and Extensive groups, relative to Controls. There was no significant change in resting metabolic rate (*p* = 0.78).

**Conclusion:**

Taken together, these results show no evidence of an “activitystat” effect. In the current study, imposed exercise stimuli of 150–300 min/week resulted in commensurate increases in overall energy expenditure and physical activity, with no sign of compensation in either of these constructs.

**Trial registration number:**

ACTRN12610000248066 (registered prospectively 24 March 2010)

## Background

Physical activity has many important physical and psychological benefits, including reducing the risk of cardiovascular disease, type II diabetes, depression and some cancers, as well as increasing life expectancy [1, 2]. In recognition of this, many countries have developed guidelines for minimum physical activity levels; however, many adults fail to meet such guidelines. Insufficient physical activity continues to be a major and costly contributor to the global burden of disease [2]. As such, efforts to increase population physical activity levels are an important preventative health measure.

A multitude of studies have been undertaken with the aim of increasing individuals’ or groups’ daily physical activity levels. Such studies have taken a variety of forms, including group-based programs, self-management programs and mass media campaigns. However, like many behaviour change interventions, physical activity interventions generally have limited success, achieving minimal or only short term change [3]. In fact, a systematic review and meta-analysis demonstrated that physical activity interventions in children had minimal effect on overall physical activity levels [4]. This review included 30 studies, with objective accelerometry data from over 6,000 participants. The level of recidivism with physical activity interventions is notoriously high, often cited at 50 % drop out after six months [5], even when the stimulus to exercise is still continuing.

One explanation that has been proposed to explain the limited success of physical activity interventions is the ‘activitystat’ hypothesis. First described in 1998 by Dr Thomas Rowland, the activitystat hypothesis suggests that when an individual increases their physical activity or energy expenditure in one domain, there is a compensatory change in another domain, in order to maintain an overall stable level of physical activity or energy expenditure [6]. Physical activity interventions typically treat physical activity as a voluntary behaviour that may be changed in a sufficiently informed and motivated individual. However, the activitystat hypothesis proposes that this mechanism is biologically regulated, with an activitystat taking on the characteristics of a homeostatic feedback loop, whereby a setpoint of physical activity or energy expenditure is maintained by compensatory adjustments through, as yet undetermined, mechanisms. It is important to clarify that the concepts of biological control of energy expenditure and the activitystat hypothesis are not co-extensive. There is considerable evidence based on rodent and human research to support the broader concept of biological control in energy expenditure regulation [7, 8], however the activitystat is a specific model of how biological mechanisms may operate using a homeostatic model. The question of if, and how an ‘activitystat’ may underpin our energy expenditure and physical activity has been actively debated in the literature [9].

Compensation, or substitution of habitual or baseline levels of activity, is not often taken into account in exercise intervention studies [10]. A systematic review of the literature has previously identified 28 studies that had experimentally investigated compensation in physical activity or energy expenditure and as such, the activitystat hypothesis [11]. The results of this review suggested that there is conflicting evidence as to the existence of an activitystat with 63, 40 and 80 % of studies involving children, adult and older adult studies respectively, reporting evidence of compensation in either physical activity or energy expenditure [11]. Several experimental papers investigating compensation have been published since this review [12–21], and similarly report conflicting results. In children and adults, several recent studies have shown some evidence of compensation with an imposed exercise stimulus [12–15], however there are at least as many that demonstrate no evidence of an activitystat or compensatory effect [16–19]. By contrast, recent studies in older adults have provided some evidence of compensation [20, 21].

A significant limitation to the current literature is that there is a lack of consistency in the methodological approaches used to investigate the activitystat hypothesis and compensation. As a result, the systematic review [11] included a number of recommendations for future studies. These included but were not limited to: measurement of both energy expenditure and physical activity using a variety of high-quality measurement tools; that activity should be assessed over sufficiently long periods and sufficiently regularly to detect compensation (with a recommendation of 4–12 weeks); that the exercise stimulus should be sufficiently high to trigger a supposed compensatory mechanism; that analyses should be ‘per protocol’ to ensure exposure to the stimulus; and finally, that a control group should be used to account for shifting baselines [11]. To date, no study comprehensively covers this methodological framework.

To address this gap the current study was specifically designed to investigate the activitystat hypothesis, taking into account these key methodological limitations. The primary aim of this study was to determine the effect of two different imposed exercise loads in previously insufficiently active adults on energy expenditure and physical activity. It was hypothesised that if an activitystat was present, then participants would adhere to the imposed exercise load, but reduce total energy expenditure and/or physical activity in other aspects of their daily life resulting in no or minimal net increase in energy expenditure of physical activity, relative to controls.

## Methods

This study used a single-blinded, multi-armed, randomised controlled trial design. Ethical approval was provided from the University of South Australia Human Research Ethics Committee and this study was registered prospectively on 24 March 2010 with the Australian and New Zealand Clinical Trials Registry (ACTRN12610000248066).

### Participants and recruitment

Using convenience sampling, potential participants were recruited via email and print advertising through a metropolitan university, a tertiary hospital and several government departments in Adelaide, South Australia. Interested participants were invited to attend an initial laboratory session to complete informed consent and the Active Australia Survey. If eligible, a second laboratory session was conducted to complete the Sports Medicine Australia Pre-Exercise Screening System. Participants who met the following inclusion criteria were invited to participate in the study: (1) aged 18–60 years at their last birthday; (2) categorised as insufficiently active, defined as participating in less than 150 min of MVPA per week according to the Active Australia Survey [22]; and (5) considered safe to start an exercise program according to the Sports Medicine Australia Pre-Exercise Screening System [23]. All participants were provided with a $200 gratuity at completion of the study.

### Measurement protocol

Participants were assessed on five measurement occasions: baseline (the week before the program began), mid- (weeks 3–4) and end-intervention (week 6), and at 3- and 6-month follow-up (weeks 12 and 24 following the intervention). Following completion of baseline testing, participants were randomly allocated to one of the three study conditions (Moderate or Extensive exercise group or a Control group) by a person external to the study using a computer-generated random allocation sequence, with allocation concealment maintained until the moment of allocation. Participants were randomised using a non-stratified, 1:1:1 allocation ratio. All outcome measures were conducted by trained research assistants who were blinded to group allocation. Although it was not possible to blind the participants to group allocation due to the nature of a physical activity intervention, participants were blinded to the activitystat hypothesis.

### Measurement tools

#### Indirect calorimetry via ventilated hood

Resting metabolic rate was measured using indirect calorimetry via a ventilated hood (ParvoMedics TrueOne 2400, ParvoMedics, Sandy, UT) at baseline and end-intervention. The measurement protocol for resting metabolic rate was developed based on a methodological review by Compher and colleagues [24]. Participants were required to be rested and fasted for a minimum of 12 h, measurements were taken in an environmentally controlled chamber with an ambient temperature of 24 °C and relative humidity of 60 % and after a 15 min equilibration period, respiratory gases were collected for 30 min. Minute ventilation, O_2_ and CO_2_ content were analysed using the ParvoMedics TrueOne analyser and minute-by-minute samples were taken to calculate resting metabolic rate. Resting metabolic rate (kcal/day) was defined as the lowest five-minute average obtained during the 30-min measurement period with a coefficient of variation of <10 % to ensure that a steady state metabolic rate was achieved [24]. The TrueOne analyser system has demonstrated reliability and validity and has been shown to yield values not significantly different from the criterion Douglas bag method [25].

#### Accelerometry

Accelerometry was used to objectively assess total activity (average total accelerometer counts per day) and physical activity (average minutes spent in moderate to vigorous physical activity per day) on all measurement occasions using the Actigraph GT3X (Actigraph, Pensacola, FL). Participants were asked to wear the accelerometer 24-h a day, for seven days at each measurement occasion except for water-based activities or contact sports. The accelerometer was initialised to capture 30-s epochs with a 30 Hz sampling frequency and was worn on an elastic waist belt on the right mid-axillary line. Participants were also asked to complete a brief wear time log during the monitoring period. A valid day was defined as a minimum wear-time of 10 waking hours, with non-wear time defined as 60 min or more of consecutive activity counts of zero. For data to be included, participants must have satisfied a minimum wear-time criteria of at least four of the seven days, one of which must have been a weekend day [26]. The Actigraph GT3X accelerometer has demonstrated acceptable intra- and inter-device reliability [27] and is considered a valid tool for estimating physical activity. Total activity (average total accelerometer counts per day) and physical activity (average minutes spent in moderate to vigorous physical activity per day) were derived from the vertical axis with a cut point of 2020 counts per minute for moderate activity [26].

#### Multimedia activity recall for children and adults

Total daily energy expenditure (MET.min) and physical activity (average minutes spent in moderate to vigorous physical activity) were measured using the Multimedia Activity Recall for Children and Adults (MARCA) [28] at all measurement occasions. The MARCA is a computerised 24-h use of time self-report recall tool that asks participants to recall everything they did in the previous 24 h from midnight to midnight, using meals as anchor points. Participants choose from over 500 discrete activities and are asked to recall activities in 5-min time intervals. Each activity in the MARCA is assigned a MET value based on an expanded version of the Compendium of Physical Activities [29], so that energy expenditure can be estimated. Originally developed for use with children, the MARCA has been modified and validated for use with adults [28]. The adult version of the MARCA has test-retest reliabilities in adults of 0.920-0.997 [28] for major activity sets such as sleep, physical activity and screen time and convergent validity between physical activity level (estimated average rate of energy expenditure) and accelerometer counts/minute of rho = 0.72 [28]. A comparison of the child and adolescent version of the MARCA with the gold standard doubly labeled water showed correlations of rho = 0.70 for total daily energy expenditure [30].

In this study, the MARCA was administered by computer-assisted telephone interview. At each measurement occasion, two separate calls (approximately 30–45 min each) were made one week apart, during which participants recalled the two previous days. At each time point participants therefore recalled four days of activity, including at least one weekday and one weekend day. For each individual participant, wherever possible, the same days of the week were recalled at each time-point. The data collection protocol for the MARCA was the same across all three groups (Control, Moderate and Extensive). Total daily energy expenditure (MET.min) was calculated using the factorial method, that is by multiplying the rate of energy expenditure associated with each activity (in METs), by the number of minutes for which that activity was performed, summing them across the day, and dividing by 1440 (minutes per day). Daily minutes spent in moderate to vigorous physical activity was derived by summing the number of minutes spent in activities likely to elicit ≥3 METs. Average physical activity level and minutes in moderate to vigorous physical activity were calculated by averaging the variables across the four recall days using a 5:2 weighting for weekday: weekend days.

### Intervention

Participants took part in either a Moderate (150 min/week) or Extensive (330 min/week) 6-week physical activity program based on a previously designed and tested physical activity intervention [31]. This program was chosen as previous studies have reported high participant compliance, providing greater assurance that there would be sufficient participation to investigate compensation. The program was a combination of aerobic and strengthening activities with progressive increases in intensity and comprised of both group-based, instructor-led exercise sessions and self-directed individual exercise sessions. The two intervention conditions involved similar types of physical activities and intensities, and differed only in volume (minutes per week). A detailed description of the exercise sessions for both conditions can be found in the published study protocol [32]. A qualified exercise physiologist conducted all group sessions and these sessions were run separately for the Moderate and Extensive groups.

The Moderate exercise intervention was designed to increase moderate to vigorous physical activity by approximately 150 min per week. Participants in the Moderate group attended instructor-led group classes three times per fortnight (60 min each). In addition, participants were also required to carry out a minimum of two self-directed sessions per week (30 min each). This dosage is consistent with the minimum level of physical activity per week recommended by Australian guidelines [33].

The Extensive exercise intervention was designed to increase moderate to vigorous physical activity by approximately 300 min per week. Participants in the Extensive group attended instructor-led group classes three times per week (60 min each). In addition, participants were also required to carry out a minimum of four self-directed sessions each week (30 min each). This dosage is consistent with the upper level of physical activity per week recommended by Australian guidelines [33].

Participants were considered ‘completers’ if they remained in the study for the duration of the intervention. To determine compliance with the prescribed exercise program, participants were provided with a purpose-designed physical activity diary (activity type, duration, mean heart rate) and a heart rate monitor (Polar 610i, Polar Electro, Kempele, Finland) which they were required to complete/use for all programmed supervised and unsupervised physical activity sessions. Participants returned the diary at the end of the intervention and the exercise physiologist downloaded participants’ heart rate monitors on a weekly basis during the 6-week program. Compliance data were entered into an Excel spreadsheet for collation prior to data analysis. Participants allocated to the control group were wait-listed for the exercise component of the program once their formal testing was completed and in the meantime were given no specific instructions.

### Statistical analysis

Statistical analyses were conducted using SPSS version 21 (IBM Corporation, Armonk, NY, United States). Participants’ demographic characteristics were analysed descriptively at baseline in accordance with the CONSORT guidelines for randomised controlled trials [34]. Differences in characteristics between completers and non-completers were analysed using Student’s t-test for continuous variables (age, body mass index and gross household income) and chi-squared tests for categorical variables (% female and group allocation). Compliance data (duration and intensity of physical activity sessions) based on objective heart rate monitoring during the intervention were analysed descriptively.

Because this study aimed to investigate the activitystat, rather than the effectiveness of the intervention, analyses were performed on a per-protocol basis where only those participants who completed the intervention were included. To address the primary aim of this study, random effects mixed modeling (using the ‘Mixed Models/Generalised Linear Models’ function and a variance components covariance structure) was used to compare the variables of interest at each time point with time (0, 3, 6, 12 and 24 weeks) and group allocation (Control vs Moderate vs Extensive) as the fixed factors. Overall group and time *p*-values are reported, in addition to group x time interaction *p*-values for each time point. When there was a significant group x time interaction effect at a given time point, post-hoc analyses with Fisher’s least significant difference tests were used to identify where the significant effect was (e.g. Control vs Moderate group, Control vs Extensive group, Moderate vs Extensive group). Post-hoc findings are indicated by superscripts in the results tables. Where the data were skewed, generalised linear mixed models were applied according to the distribution. A significant group by time interaction indicated a significant difference in energy expenditure or physical activity among the groups. Alpha was set at 0.05. While no correction has been made for multiple comparisons, actual *p*-values are reported. A priori power calculations determined that a sample of 36 participants per group (*n* = 108) would be sufficient to detect small effect sizes (Cohen’s d ≥ 0.3) for measurements taken five times and small to moderate effect sizes (Cohen’s d ≥ 0.4) for measurement taken twice, at 5 % alpha and 80 % power.

## Results

### Participants and compliance

**Fig. 1:**
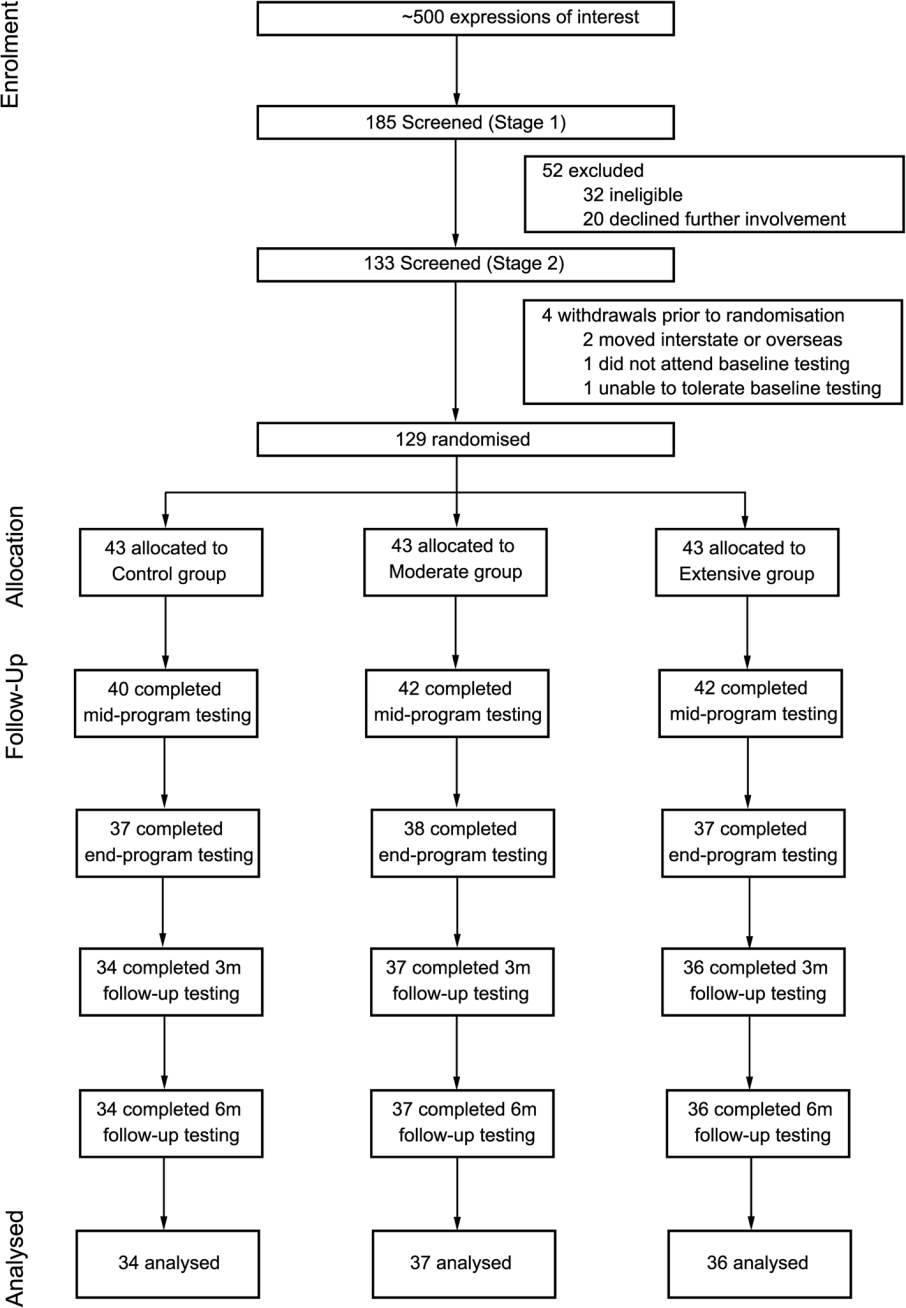
CONSORT flow diagram of participant recruitment, enrolment and progression through the study

A total of 129 participants completed baseline testing and were randomly allocated to the Control (*n* = 43), Moderate (*n* = 43) or Extensive group (*n* = 43). See Fig. 1 for a CONSORT flow diagram demonstrating details of participant recruitment, enrolment and progression through the study. A total of *n* = 107 participants completed the study with *n* = 22 participants withdrawing [Control (*n* = 9), Moderate (*n* = 6), Extensive (*n* = 7], resulting in an overall retention rate of 83 %. Reasons for withdrawal included being unable to commit the time required for the study [*n* = 11; Control (*n* = 8), Moderate (*n* = 3, Extensive *n* = 0)], personal, work or family reasons [*n* = 7; Control (*n* = 1), Moderate (*n* = 2), Extensive (*n* = 4)] or medical reasons, unrelated to the physical activity program [*n* = 4; Control (*n* = 0), Moderate (*n* = 1), Extensive (*n* = 3)]. Descriptive summary statistics for sociodemographic and anthropometric variables for the whole sample and for completers only are provided in Table 1. Most were in full employment in mainly professional or clerical positions, 64 % were women, and they came from households that were economically advantaged relative to the general Australian population. Baseline characteristics were not formally tested for differences in accordance with the 2010 CONSORT statement [34]. Completers were more likely to be older (*p* < 0.01; mean age of 43 years compared to 33 years for the non-completers). There was no statistical difference between completers and non-completers for gender (*p* = 0.22), gross household income (*p* = 0.88), body mass index (*p* = 0.74) or group allocation (Control, Moderate, Extensive; *p* = 0.68). The average (SD) number of valid days for accelerometry was 8.0 (2.31) days for baseline, 7.3 (0.91) days for mid-intervention, 7.4 (0.73) days for end-intervention, 7.6 (1.06) days for 3-month follow-up and 7.5 (1.0) days for 6-month follow up. Average (SD) wear time across valid days was 24.0 (0.02) h for baseline, 23.9 (0.25) h for mid-intervention, 23.9 (0.13) h for end-intervention, 24.0 (0.12) h for 3-month follow-up and 24 (0.07) h for 6-month follow-up.

**Table 1:** Baseline sociodemographic and anthropometric characteristics of the whole sample (*N* = 129) and completers (*N* = 107)

	Whole sample	Control	Moderate	Extensive
N	129	43	43	43
Age (years)	41 (11)	40 (11)	41 (11)	43 (11)
Height (cm)	169.2 (8.8)	169.7 (8.9)	170.0 (8.8)	167.8 (8.7)
Weight (kg)	80.3 (17.6)	79.7 (20.7)	79.1 (15.6)	82.2 (16.3)
Body mass index (kg/m^2^)	26.1 (5.2)	25.8 (6.1)	25.6 (4.8)	27.0 (4.5)
Household income^a^	102 (47)	104 (53)	104 (37)	97 (50)
% Female	66	60	67	70
	Completers	Control	Moderate	Extensive
N	107	34	37	36
Age (years)	43 (11)	43 (10)	41 (12)	45 (10)
Height (cm)	169.3 (8.5)	169.7 (9.2)	169.6 (7.6)	168.6 (9.0)
Weight (kg)	80.2 (17.1)	79.9 (20.5)	78.8 (15.6)	81.7 (15.1)
Body mass index (kg/m^2^)	26.2 (5.0)	26.1 (5.8)	25.7 (5.1)	26.8 (3.9)
Household income^a^	102 (42)	104 (52)	104 (33)	97 (42)
% Female	64	59	68	64

*Note:* Unless otherwise indicated, values are mean (standard deviation). ^a^Pre-tax income in thousands of Australian dollars per annum. N = sample size

Compliance with the prescribed physical activity program was measured by the frequency, duration and intensity of sessions recorded by objective heart rate monitoring. In accordance with the per protocol analysis, the following compliance data are presented for completers only (*n* = 37 Moderate group; *n* = 36 Extensive group). Over the 6-week intervention, participants recorded an average total of 13 sessions in the Moderate group and 33 sessions in the Extensive group. On average, the weekly duration of recorded sessions was 195 ± 63 min/week in the Moderate group and 386 ± 40 min/week in the Extensive group. Group sessions accounted for an average of 97 ± 56 min/week and 172 ± 44 min/week of the total duration in the Moderate and Extensive groups, respectively. Intensity was determined on the basis of average heart rate for the entire recorded session (as a percentage of age-predicted maximal heart rate) for each participant. This included time spent in warm up and stretching activities and the cool-down period. Average weekly intensity in both groups ranged from approximately 65–75 % HRmax.

### Main findings

The constructs of energy expenditure and physical activity were measured in multiple ways using a combination of variables and measurement tools. The construct of energy expenditure included self-reported total daily energy expenditure measured using the Multimedia Activity Recall for Children and Adults, total activity measured using accelerometry and resting metabolic rate measured using indirect calorimetry. The construct of physical activity was defined as moderate to vigorous physical activity and was objectively measured using accelerometry and self-reported using the Multimedia Activity Recall for Children and Adults.

#### Energy expenditure

**Fig. 2:**
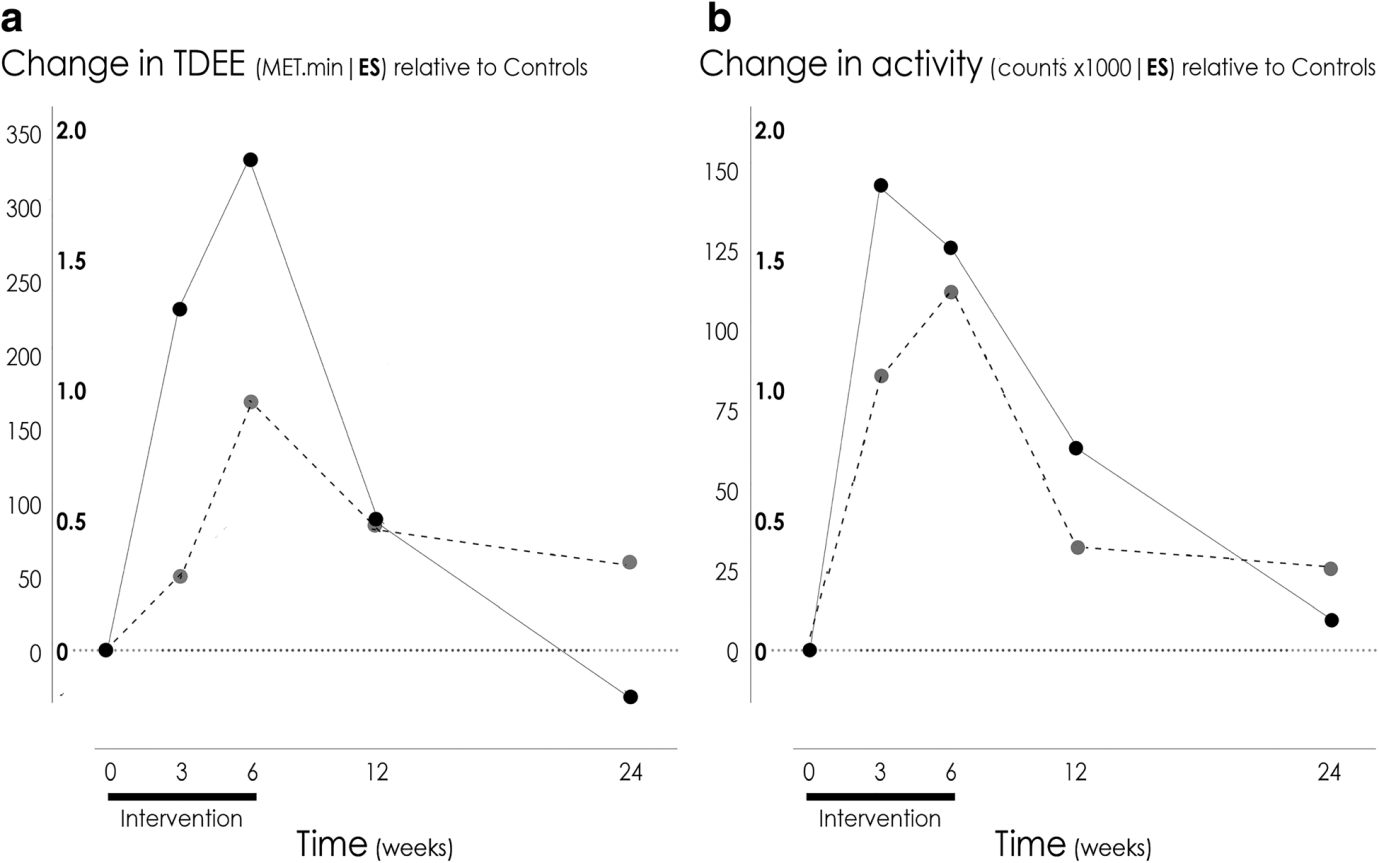
Change in total daily energy expenditure (MET.min; measured by the MARCA) (Panel **a**) and total activity (total counts/day; measured by accelerometry) (Panel **b**) in the Moderate (− − −) and Extensive (―) groups. Data are presented as effect sizes, expressed as change relative to the Control group, divided by the pooled SD at baseline (across all three groups). The outer units on the y-axis represent the scale of the change in the original units. *Note:* TDEE = total daily energy expenditure; MARCA = Multimedia Activity Recall for Children and Adults; ES = effect size; SD = standard deviation

Random effects mixed modeling analyses and raw descriptive statistics for the construct energy expenditure are presented in Table 2. There was a consistent pattern of an increase in energy expenditure in the Moderate and Extensive groups during the intervention, relative to the Control group. This trend reached significance in both accelerometry and MARCA estimates (*p* < 0.001). No matter how energy expenditure was measured, the Extensive group showed a greater increase than the Moderate group at the end of the 6-week intervention. Following the conclusion of the intervention, there was also a consistent pattern for energy expenditure to decrease. While some outcomes remained significantly different from baseline at three month follow up (total activity; *p* = 0.02), no variables were significantly different from baseline at six-month follow up. Resting metabolic rate did not significantly change across groups over time. Figure 2 demonstrates the magnitude of change in the outcome variables over time. Data are presented as effect sizes, expressed as changes in the intervention groups (relative to change in the Control group), divided by the pooled standard deviation at baseline across all three groups. All changes are calculated from baseline.

**Table 2:** Construct: Energy expenditure; results of random effects mixed modeling analysis

Outcome	Measure (unit)	Time	Control	Moderate	Extensive	P	P	P
			Mean (SD)	Mean (SD)	Mean (SD)	Group	Time	Group x Time
TDEE	MARCA	Overall				**<0.001**	**<0.001**	**<0.001**
	(MET.min)	Baseline	2,160 (121)	2,155 (205)	2,190 (194)			0.67
	Mid	2,178 (169)^a^	2,222 (176)^b^	2,432 (230) ^ab^				**<0.001**
	End	2,127 (181) ^a^	2,286 (249) ^a^	2,491 (216) ^a^				**<0.001**
	3 month	2,178 (194)	2,263 (263)	2,301 (280)				0.11
	6 month	2,208 (167)	2,258 (270)	2,207 (251)				0.56
Total activity	ACC	Overall				**<0.001**	**<0.001**	**<0.001**
	(total counts/day)	Baseline	265,913	252,453	269,443			0.61
			(94,299)	(79,978)	(66,943)			
	Mid		257,978	327,353	406,693			**<0.001**
			(76,309)^a^	(91,290)^a^	(90,813)^a^			
	End		256,346	354,139	386,223			**<0.001**
	3 month		245,060	263,087	312,147			**0.01**
			(65,983)^a^	(95,248)^b^	(83, 508)^ab^			
	6 month		252,308	264,777	263,456			0.85
			(91,623)	(92,075)	(94,913)			
RMR	IC	Overall				0.86	**<0.001**	0.34
	(kcal/day)	Baseline	1,509 (240)	1,505 (227)	1,536 (260)			
		End	1,541 (244)	1,576 (231)	1,584 (284)			

*Note:* Summary data are raw scores and significant differences indicated in bold. Values with the same superscript were significantly different on post-hoc analysis

MARCA variables: *N*=107 (Control, *n*=34, Moderate, *n*=37, Extensive, *n*=36). Accelerometry variables: *N*=95 (Control, *n*=28, Moderate, *n*=35, Extensive, *n*=32)

Indirect calorimetry variables: *N*= 94 (Control, *n*=29, Moderate, *n*=34, Extensive, *n*=31)

*TDEE *total daily energy expenditure (MET.min), *MARCA* Multimedia Activity Recall for Children and Adults, *RMR* resting metabolic rate (kcal/day), *IC* indirect calorimetry, *ACC* accelerometery, *SD* standard deviation

#### Physical activity

**Fig. 3:**
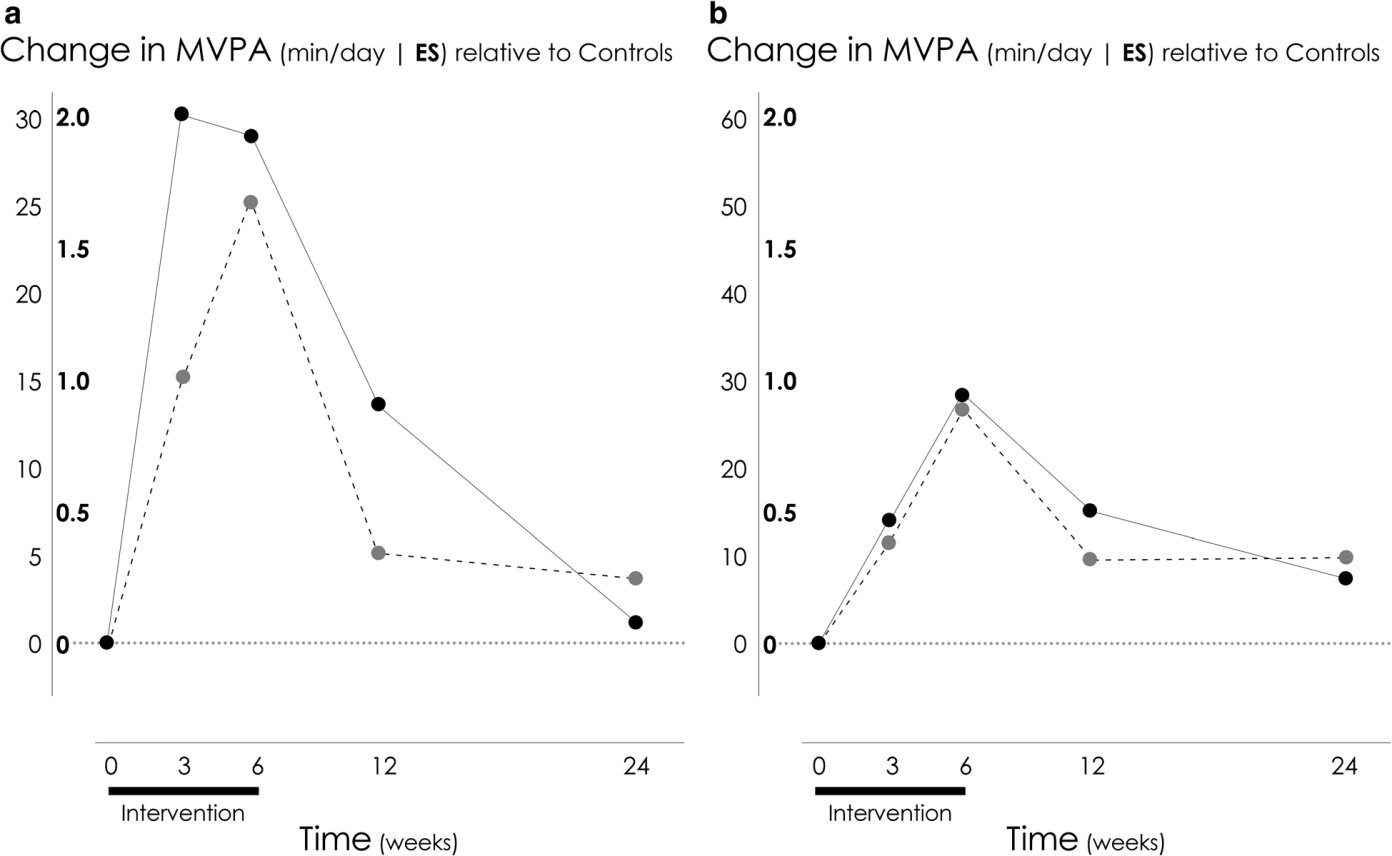
Change in moderate to vigorous physical activity (min/day) measured by accelerometry (Panel **a**) and MARCA (Panel **b**) in the Moderate (− − −) and Extensive (―) groups. Data are presented as effect sizes, expressed as change relative to the Control group, divided by the pooled SD at baseline (across all three groups). The outer units on the y-axis represent the scale of the change in the original units. *Note:* MVPA = moderate to vigorous physical activity; MARCA = Multimedia Activity Recall for Children and Adults; SD = standard deviation; ES = effect size

Random effects mixed modeling analyses and raw descriptive statistics for the construct physical activity are presented in Table 3. Similar trends were seen in changes in moderate to vigorous physical activity, regardless of the method of measurement, although MARCA mean estimates were consistently higher than those determined by accelerometry. Minutes spent in moderate to vigorous physical activity significantly increased in the Moderate and Extensive groups compared to the Control group across the intervention period, according to both accelerometry and MARCA estimates (*p* < 0.001). Following the conclusion of the intervention, moderate to vigorous physical activity declined, although remained statistically elevated at three months according to accelerometry (*p* = 0.02). No significant difference was seen in either method of measurement at six month follow up. Figure 3 demonstrates the magnitude of change in the outcome variables over time. Data are presented as effect sizes, expressed as changes in the intervention groups (relative to change in the Control group), divided by the pooled standard deviation at baseline across all three groups. All changes are calculated from baseline.

**Table 3:** Construct: Physical activity; results of random effects mixed modeling analysis

Outcome	Measure	Time	Control	Moderate	Extensive	P	P	P
			Mean (SD)	Mean (SD)	Mean (SD)	Group	Time	Group x Time
MVPA	ACC	Overall				**<0.001**	**<0.001**	**<0.001**
	(min/day)	Baseline	34 (16)	34 (14)	35 (11)			0.91
		Mid	35 (15)^a^	48 (15)^a^	64 (16)^a^			**<0.001**
		End	32 (14)^ab^	55 (17)^a^	59 (23)^b^			**<0.001**
		3 month	31 (13)^a^	36 (17)	43 (16)^a^			**0.03**
		6 month	34 (19)	37 (17)	36 (16)			0.70
MVPA	MARCA	Overall				**0.02**	0.06	**0.01**
	(min/day)	Baseline	111 (47)	108 (50)	120 (69)			0.70
		Mid	112 (56)	129 (79)	147 (51)			0.07
		End	99 (48)^ab^	143 (74)^a^	159 (56)^b^			**<0.001**
		3 month	109 (53)^a^	121 (70)^b^	148 (90)^ab^			**0.05**
		6 month	116 (58)	129 (70)	112 (70)			0.49

*Note:* Summary data are raw scores and significant differences indicated in bold. Values with the same superscript were significantly different on post-hoc analysis

MARCA variables: *N*=107 (Control, *n*=34, Moderate, *n*=37, Extensive, *n*=36). Accelerometry variables: *N*=95 (Control, *n*=28, Moderate, *n*=35, Extensive, *n*=32)

*MVPA* moderate to vigorous physical activity, *MARCA* Multimedia Activity Recall for Children and Adults, *ACC* accelerometery, *SD* standard deviation

## Discussion

The primary aim of this study was to test the activitystat hypothesis by determining the effect of two six-week physical activity interventions, of differing volumes, on energy expenditure and physical activity in previously inactive, healthy adults. The hypothesis was that if an activitystat was present, participants would adhere to the imposed exercise load, but reduce total energy expenditure and/or physical activity in other aspects of their daily life in order to achieve no or minimal net increase in energy expenditure of physical activity, relative to controls. The results of the study did not support the existence of an activitystat. At end-intervention, significant increases in energy expenditure and physical activity were demonstrated in the Moderate and Extensive groups, relative to Control. These increases were either in excess or largely commensurate with the respective imposed exercise loads (150 or 300 min per week in the Moderate and Extensive groups, respectively). No significant changes were demonstrated in resting metabolic rate at end-intervention. At 6-month follow up, all energy expenditure and physical activity variables were non-significant between groups and had returned to baseline levels.

### Main findings

To find evidence of an activitystat, it would have been necessary to demonstrate that the intervention groups compensated for the imposed exercise stimulus, relative to the Control group. Compensation could occur through regulation of overall energy expenditure (i.e. compensatory changes in resting metabolic rate or through the spectrum of activity intensities – sedentary, light, moderate and vigorous) or through moderate to vigorous physical activity alone (i.e. direct exchange of habitual moderate to vigorous physical activity for imposed moderate to vigorous physical activity) [11]. If energy expenditure was the regulated variable within the activitystat, there should be no or minimal net increase in energy expenditure with the intervention. Similarly, if physical activity was the regulated variable, programmed moderate to vigorous physical activity would be exchanged for non-programmed moderate to vigorous physical activity, again resulting in no or minimal net increase in physical activity with the imposed stimulus. The current study found no evidence for the existence of the activitystat in this population. That is, there was an increase in both energy expenditure and physical activity with a programmed exercise intervention and no evidence of compensation either in non-programmed activity or in resting metabolic rate. These findings were consistent across multiple outcome measures.

These findings are consistent with a recent systematic review on this topic [11]. In this review, 15 studies were identified that investigated compensation in energy expenditure or physical activity in response to an exercise interventions in an adult population. The majority of these studies (9/15) did not support compensation, and therefore did not support the activitystat hypothesis. Only one study specifically set out to test the activitystat hypothesis [35], and while they also did not find any evidence of its existence, their methodology is not comparable. Dale and colleagues [35] investigated compensation in physical activity measured by accelerometry in children rather than adults, and used a cross-over design where children acted as their own controls, rather than a randomized controlled trial. Furthermore, instead of imposing a stimulus of increased physical activity, they restricted activity in school breaks, hypothesising that this would be compensated by an increase in activity after school.

Since the systematic review, several additional studies have been published investigating the broader concept of compensation with an imposed exercise stimulus. Our results are consistent with the findings of Kozey-Keadle and colleagues [13], who failed to identify any differences in non-exercise MVPA and total activity (MET.hrs) between an exercise and control group with a 12-week moderate exercise intervention. Similar to the current study, Kozey Keadle et al. used a randomised controlled trial design with an objective and frequent measure of physical activity and overall energy expenditure. The participants were similar in age, activity and status (aged 20–60 years, previously inactive but otherwise healthy), however had a higher BMI (average BMI 35.1 kg/m^2^ compared to 26.2 kg/m^2^) compared to the current study.

In contrast, Wasenius and colleagues [12] demonstrated no significant increase in total leisure time physical activity with either a Nordic walking or resistance training exercise intervention compared to a control group. While Wasenius et al. also used a randomised controlled trial design with two different interventions, there were several key methodological differences to the current study. Wasenius and colleagues used a sample of overweight men who were clinically at an increased risk of type II diabetes. In addition, the study design did not include an objective measure of activity or, in fact, any measure of activity outside of purposeful physical activity sessions lasting more than 30 min. Physical activity data were collected by self-report diary where participants were required to record the duration, type and intensity. These entries were then converted to METs using a database of known energy costs. It is likely that energy expenditure and physical activity were significantly underestimated as no incidental activity or any activities lasting <30 min were captured using this method.

### Plausibility of the activitystat hypothesis

The impetus behind this study grew out of recent discussions of the activitystat hypothesis in the physical activity literature. Open debate about an activitystat has revealed widely divergent views by researchers in the field. It is a novel hypothesis, and support for its existence has grown primarily from observational studies in children, and a small number of frequently cited experimental papers in adolescents, adults and older adults that have demonstrated less than expected increases in energy expenditure or physical activity with an imposed exercise stimulus.

There are plausible a priori arguments to support the notion of an activitystat. These reasons include; the strong pattern of recidivism evident with physical activity interventions, often when the program is still running [3]; observational studies demonstrating consistency of physical activity independent of environment or opportunity [36]; and a growing evidence base in animal and human research to support biological determinants of physical activity [7]. However, there are also several a priori reasons why biological control of energy expenditure or physical activity is not likely to take on the specific functional model proposed in the activitystat hypothesis.

Firstly, the activitystat hypothesis is concerned exclusively with the regulation of energy expenditure or physical activity. There are good evolutionary reasons why energy balance and energy stores may be regulated for survival [37], but to control them using only energy expenditure would seem inefficient. It is more likely that energy expenditure and physical activity would be regulated in the context of energy intake and body weight maintenance [38, 39]. For example, there is evidence to suggest that energy balance is more effectively maintained at higher levels of energy expenditure and energy intake [40]. Energy output has always been intimately linked with energy intake based on research findings of human ancestral studies. In an environment where physical activity was obligatory and where energy scarcity not uncommon, natural selection ensured that those who could maintain energy balance survived. Despite the changing face of modern behaviour and environments, this relationship has contributed to the genetic profile of humans in the 21^st^ century [41]. In fact, many intervention studies interpret energy expenditure or physical activity compensation in light of energy balance. For example, weight loss is often less than expected based on the potential energy deficit from the programmed exercise [42]. Measurement of physical activity or energy expenditure is often used in these studies to determine whether the predicted energy expenditure was compensated in non-programmed energy expenditure or whether there was a compensatory increase in energy intake. However, it is important that when non-programmed energy expenditure is calculated in these studies, it is done so against the background of baseline or habitual physical activity in order to capture substitution of habitual physical activity for the imposed exercise program [10].

Secondly, when individuals start an exercise program, the time to participate in the program (i.e. the actual time spent in additional physical activity) and the collateral time (i.e. additional travel and self care) must be drawn from somewhere. One proposition of the activitystat hypothesis is that the time will be drawn from baseline or habitual moderate to vigorous physical activity, resulting in no net increase in overall energy expenditure or physical activity. In fact, there is good reason to believe that the time will actually be drawn from discretionary activities with large volumes of time already devoted to them, for example, television viewing. The most recent Australian Bureau of Statistics Time Use Survey reports that, on average, Australians spend 179 min/day watching television, accounting for just over 12 % of a total day [43]. In fact, insufficiently active populations by definition are participating in, on average, less than 20 min/day of programmed physical activity, accounting for only 1.4 % of a total day. In the current study we imposed a load of 1.5–3 % of the day in moderate to vigorous physical activity. At baseline, participants, while not meeting the activity guidelines, were still participating in moderate to vigorous physical activity for around 8 % per day, if a whole-of-day approach is taken to determine activity levels (i.e. incorporating chores etc.). This means that while there is a theoretical buffer available for compensation in moderate to vigorous physical activity, it is more likely to come from sedentary or light intensity activities that account for larger proportions of the day.

In addition to these plausible reasons why an activitystat may or may not exist, critical examination of the literature reveals very mixed results [11]. Existing studies measuring compensation vary widely, making synthesis difficult. Most notably, the current literature is limited by a lack of studies that have specifically set out to experimentally test the activitystat hypothesis using purpose-designed approaches. Instead, many of the studies that directly reference an activitystat are secondary analyses of cross-sectional studies, where conclusions are limited to associations and causality of compensation cannot be demonstrated. The current study aimed to address these methodological limitations by investigating the activitystat hypothesis using a purpose- designed, randomised controlled trial, the highest level of primary evidence and did not find evidence of an activitystat in an adult population.

Energy expenditure and physical activity outcomes measured by the MARCA and accelerometry indicated a downward trend at 3- and 6-month follow up compared with the end of the program, with all outcomes returning to baseline levels at six months in the intervention groups. A follow-up period was included in this study because it was important to monitor how the regulated variables respond once the stimulus is removed, particularly if compensation had been detected. Some may argue that the return to baseline after 3–6 months reflects a loosely defended set point of energy expenditure or physical activity and is therefore evident of an activitystat. However, without evidence of compensation while the stimulus was applied, the conditions of the specific mechanism of the activitystat are not satisfied [6]. Further, behavioural and environmental drivers for this return to baseline cannot be discounted using this methodological design.

### Strengths and limitations

A key strength of the current study was that it was designed specifically to test the activitystat hypothesis. In doing so, the design of the study took into account the considerations unique to measuring an activitystat [11]. The current study clearly defined that the regulated variable could be either energy expenditure or physical activity, and used a triangulation of three independent measures (accelerometry, self-report and indirect calorimetry) to measure these variables and their components. It also employed an intervention incorporating both group and individual exercise sessions with a duration that should theoretically be sufficiently long enough to induce compensation, while measuring the regulated variables regularly both during and after the stimulus to ensure that the time course of any changes was captured. Finally, the inclusion of two intervention arms, with differing doses of physical activity, allowed the tolerance of the system to be investigated.

Additionally, in order to interpret the results of a study in the context of the activitystat hypothesis, exposure to the stimulus must be confirmed before drawing conclusions about compensatory behaviors. This study objectively measured compliance with the intervention conditions, both supervised and unsupervised, and employed a per protocol analysis strategy, confirming exposure to the prescribed physical activity stimulus and strengthening confidence in its conclusions. It also used a randomized controlled trial design, the highest level of primary research evidence.

Several limitations to the current study must be acknowledged. Firstly, the generalizability of the results is limited due to the study’s sample of middle-aged, socio-economically advantaged participants from one Australian city. Secondly, it could be argued that recruiting participants who were insufficiently active reduced the opportunity for compensation to occur. According to baseline accelerometry assessments, participants recruited for this study had average levels of moderate to vigorous physical activity (34 min/day), comparable to those reported in the NHANES study [44], and sufficient for at least partial compensation in this construct (compensation in light physical activity would have been captured within the energy expenditure construct). Participants with average levels of moderate to vigorous physical activity were specifically targeted because evidence of an activitystat has important health implications in this population. If participants had high levels of physical activity at baseline, opportunity to increase physical activity would have been limited, which was necessary for compensation to have occurred, and the public health implications would have been marginal. Thirdly, the study is limited by the lack of a gold standard measure of total energy expenditure. A high-resolution measure of energy expenditure such as doubly labeled water would have also captured the smaller changes in energy expenditure that may contribute to compensatory responses (e.g. fidgeting). Finally, the tool used for screening potential participants, the Active Australia Survey, has shown limited convergent validity when compared to accelerometry (correlation coefficient rho = 0.25-0.29, *p* < 0.005) [22]. In fact, as previously mentioned, according to baseline assessments participants deemed inactive by the Active Australia survey were accruing an average of 34 min/day (241 min/week) of moderate to vigorous physical activity measured by accelerometry, and considerably more when measured by self-report. This volume is well over the lower threshold of the ‘active’ classification of 150 min of moderate to vigorous physical activity per week [33]. The limited validity of this tool is likely due to its self-report nature, allowing participants to manipulate responses to advertised eligibility criteria and to the fact that it does not take into account activities such as gardening and housework, now commonly accepted as sources of moderate to vigorous physical activity [29].

### Directions for future research

The current study has highlighted a number of areas of consideration for future studies investigating the activitystat hypothesis and physical activity interventions. Firstly, this study has rigorously investigated the activitystat in a previously inactive, adult population using a mixed aerobic and resistance exercise intervention of varying volumes. Coupled with the results of our systematic review of this topic [11], this study provides evidence to refute the existence of the activitystat in this population and under these intervention conditions. However, it is possible that an activitystat may operate under more challenging intervention conditions or in other groups such as children and older adults. While there is limited high-quality evidence to support or refute the hypothesis in these populations, there are feasible reasons why it may be more plausible in these groups. For example, children have higher levels of physical activity to trade off against an imposed physical activity stimulus, while older adults typically have lower activity levels and may have less capacity to tolerate an imposed stimulus, even temporarily [45]. Future research should focus on developing high quality experimental studies to build on the existing observational and methodologically limited experimental activitystat and compensation literature in these populations.

## Conclusions

This study has demonstrated no evidence for an activitystat in previously inactive, but healthy, adults. The activitystat is an important hypothesis to test because it suggests that standard interventions to increase physical activity may be ineffective. In its current form, the activitystat hypothesis suggests energy expenditure or physical activity is determined exclusively by biological determinants, irrespective of other variables and unable to be changed with intervention. Therefore, the implications of accepting this hypothesis without sufficient evidence is profound, given the well documented risks of physical inactivity [2]. While future research may continue to shed light on this debate, physical activity should continue to be prioritised as a means of promoting health, in the absence of sufficient evidence to suggest otherwise.

## Abbreviations

Kcal: Kilocalories
MARCA: Multimedia activity recall for children and adults
METs: Metabolic equivalents
min: Minutes
